# Recovery after Taking and Retaining Two Hundred and Eighty Grains of Chloral and Two Grains of Morphia

**Published:** 1882-02

**Authors:** R. M. Griswold

**Affiliations:** Member and Fellow of the Connecticut Medical Society, North Manchester, Conn.


### RECOVERY AFTER TAKING AND RETAINING TWO HUNDRED AND EIGHTY GRAINS OF CHLORAL AND TWO GRAINS OF MORPHIA.

By R. M. GRISWOLD, M. D.,
Member and Fellow of the Connecticut Medical Society, North Manchester, Conn.

At 10 P. M. of November 24th, 1881, I saw a young lady, aged about 30, who for several days had been laboring under considerable mental disturbance, immediately occasioned by a severe odontalgia. She had been under my professional care for the past year and a half for varied troubles.

First, for a very severe cerebro-spinal meningitis, from which her recovery seemed extremely doubtful. Afterward for uterine displacement, and a chronic cystitis, with hystero-epilepsy, the latter probably a sequel of the meningitis. Before coming into my hands she had acquired the habit of taking morphia in doses of from onequarter to one-third of a grain, twice a day, and during the whole of the past year, her mental equilibrium had been hanging in the balance. On the date mentioned I found her wandering about the house after having made several ineffectual attempts to get out of doors. After some time I prevailed upon her to allow her attendants to undress and put her to bed and soon after I gave her forty grains of chloral with one-quarter grain of sulphate of morphia. Immediately after swallowing it, she threw her arms above her head and went into a hysterical convulsion. These having previously been of so frequent occurrence upon any slight cause, I soon left her, believing she would shortly become quiet, and sleep be induced by the chloral, as had frequently been the case before.

At 6 A. M. of the next day I was summoned to see her, the messenger saying she was unconscious and they were unable to arouse her. I found her rigid, no pulse at the wrist, extremities very cold, pupils contracted to the size of a pin-head, respiration hardly discernible, and but about eight per minute, and temperature by thermometer 94½°. Her face was flushed and eyelids swollen, lower jaw dropped, tongue protruding, dry and swollen, and head drawn back between the shoulders, her mouth was filled with a white froth, and half an hour before she had vomited about a gill of very dark, thick gummy matter. She had not vomited previously. Her appearance and every symptom indicated a fatal dose of chloral, but I could not believe the forty grains I had given her the evening before sufficient to have produced such a result, under the circumstances. As it was impossible to introduce any thing into her stomach, and my hypodermic syringe having been broken the day before I ordered bottles of hot water to her feet, an ice bag to her head, and applied electricity from a Gaiffe battery, a pole under each arm, with thorough hand rubbing at the same time over the whole body, and flannels wet in hot mustard water over the heart. In three hours a slight flutter could be felt at the wrist and the temperature had risen to 96½°, respiration 12. By persistent use of the battery, and rubbing for twenty-four hours, her temperature rose to normal, respirations to 15, and at the end of that time she apparently was slightly conscious and swallowed two teaspoonfuls of wine, with the 1-16th grain of strychnia, and from this time gradually recovered from the influence of the drug, with no bad effects.

In the September previous, I had prepared for her with my own hands a mixture containing chloral ℥ i, sulphate morphia, grs. iv, to be given in twelve doses, one each night. At that time six doses had been given, when it was discontinued, the bottle conspicuously marked "poison," and placed in a closet in her bed-room. On the night in question, she had secreted this bottle about her person, and during the temporary absence of her attendant from the room, and while I was in the next room, had taken the whole amount, and in a few minutes after, suspecting nothing of what had occurred, I had given her forty grains of chloral, and one-quarter grain of morphia additional. She afterward confessed to me that this was the case, and that she did it with suicidal intent. I relate the case, because so far as I have been able to ascertain it is the only one recorded where so large amounts of these drugs combined, have been taken and retained, or where so large a quantity of chloral has been taken alone and death has not followed. The largest amount taken, followed by recovery, of which I can find any record is mentioned by Taylor in his "Treatise on Poisons," where on page 617, he speaks of recovery after doses of 150 to 160 grains.

